# Expanding the Versatility of Phage Display I: Efficient Display of Peptide-Tags on Protein VII of the Filamentous Phage

**DOI:** 10.1371/journal.pone.0014702

**Published:** 2011-02-24

**Authors:** Geir Åge Løset, Bjarne Bogen, Inger Sandlie

**Affiliations:** 1 Centre for Immune Regulation, University of Oslo, Oslo, Norway; 2 Department of Molecular Biosciences, University of Oslo, Oslo, Norway; 3 Institute of Immunology, University of Oslo, Oslo, Norway; University of Southampton, United Kingdom

## Abstract

**Background:**

Phage display is a platform for selection of specific binding molecules and this is a clear-cut motivation for increasing its performance. Polypeptides are normally displayed as fusions to the major coat protein VIII (pVIII), or the minor coat protein III (pIII). Display on other coat proteins such as pVII allows for display of heterologous peptide sequences on the virions in addition to those displayed on pIII and pVIII. In addition, pVII display is an alternative to pIII or pVIII display.

**Methodology/Principal Findings:**

Here we demonstrate how standard pIII or pVIII display phagemids are complemented with a helper phage which supports production of virions that are tagged with octa FLAG, HIS_6_ or AviTag on pVII. The periplasmic signal sequence required for pIII and pVIII display, and which has been added to pVII in earlier studies, is omitted altogether.

**Conclusions/Significance:**

Tagging on pVII is an important and very useful add-on feature to standard pIII and pVII display. Any phagemid bearing a protein of interest on either pIII or pVIII can be tagged with any of the tags depending simply on choice of helper phage. We show in this paper how such tags may be utilized for immobilization and separation as well as purification and detection of monoclonal and polyclonal phage populations.

## Introduction

Phage display is a platform for selection of binders with affinity for specific target molecules, and also exhibits high versatility with respect to target discovery [1]. In both cases, libraries of polypeptides are created as fusions to phage coat proteins that are solvent exposed [2], [3].The wt filamentous phage virions M13, fd and f1 have about 2,700 copies of the major coat protein pVIII, and in addition, express approximately 3–5 copies each of pIII, pVI, pVII and pIX; pIII and pVI on one virion tip and pVII and pIX on the other [4]. Polypeptides have been fused to and displayed on all five structural proteins, but only pIII and pVIII display have gained widespread use. With the exception of pVI display, which has been evaluated for use with cDNA libraries [5], it is common to the majority of phage display protocols that the heterologous peptide is placed *in-frame* between an *N*-terminal signal sequence and the mature form of the viral capsid protein. Alternatively, the heterologous peptide can be combined with a modified version of pIII in the periplasm, but both components are still dependent on signal sequence-directed periplasmic targeting [6], [7].

Fusion coat proteins may be encoded either in a phage genome or by a phagemid, and in the latter case, complementation by a helper phage is needed to support virion production.

It is also possible to combine multiple display of more than one type of fusion protein per virion by using both genome-based vectors [8], [9], [10], [11], [12] and phagemid systems [13]. The resulting bifunctional phage particles have been utilized in a number of areas such as life sciences [9], as therapeutics [8], [10], [11] and in solid state material sciences [12], [13].

Since pVII, like pIII, is found at the virion tip in 3–5 copies, peptide display on pVII might be an attractive alternative to pIII display, and indeed, genomic pVII display has been reported [14]. In that study, an *N*-terminal signal sequence targeting the fusion protein to the periplasm was held as a component critical for successful display. However, of the five structural capsid proteins that build the filamentous phage virion, only pIII and pVIII are synthesized as precursors containing *N*-terminal signal sequences [4].

Here, for the first time we demonstrate that signal sequence directed periplasmic targeting of the fusion is not necessary for functional pVII display. We explore display through pVII and engineer helper phage genomes that combined with standard pIII or pVIII phagemid display support the production of bispecific virions. Defined tags were added to virions with pIII or pVIII fusions. Thus, we used the modified helper phages to effectively FLAG-, HIS_6_- and AviTag-tag phage particles with pIII or pVIII phagemid-encoded fusions. We also show pIII/pVII phage-genome double display. Thus, we make existing phage display technology easily compatible with an extensive molecular biology tool box for immobilization, purification and detection.

## Results

### M13 Helper Phages with Tags Fused to pVII

**Figure 1:**
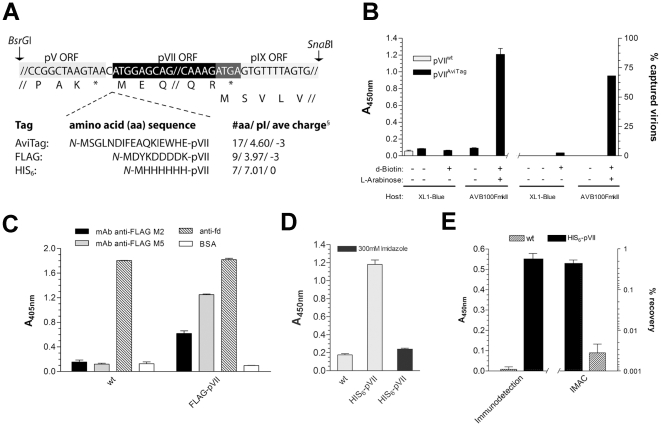
pVII tag-modified helper phages are structurally and functionally identical to normal helper phages, and donate a defined phenotype to pVII. (**A**) Schematic illustration of the pV, pVII, pIX genomic junctions in the M13 genome framed by the unique *BsrG*I/*SnaB*I RE sites. The three tag modifications, their site of insertion and physical characteristics are given. Isoelectric point (pI) as well as average charge (§) was computed using ProtParam (http://ca.expasy.org/). (**B**) AviTag-pVII functionality as BirA substrate assessed by virion binding to magnetic SA beads and detection with an anti-M13 Ab (BirA enzyme activity provided by *E. coli* XL1-Blue or AVB100FmkII). (**C**) FLAG-pVII functionality as assessed by virion binding to immobilized anti-FLAG M2 and M5 mAbs and detection with anti-M13 Ab. (**D**) HIS_6_-pVII functionality assessed by virion binding to magnetic IMAC beads and detection with an anti-M13 Ab. Virion binding to the beads was done without (*gray bars*) or with (*black bar*) 300 mM imidazole in the binding buffer. (**E**) HIS_6_-pVII functionality assessed as in D, employing HIS_6_-pVII tagged pVIII phagemid display (pGALD8mFN, *unpublished*) virions (*left part*). Specific IMAC bead capture efficiency assessed by titration of imidazole-eluted pVIII phagemid virions after bead capture, shown as percent of in-put (% recovery = out-put (cfu^ampR^)/in-put (cfu^ampR^)×100), *right part*.

A series of mutants were constructed based on the two genetically different, but functionally identical helper phage genomes, M13K07 and VCSM13. We inserted either an octa-FLAG, hexa-His (HIS_6_) or a BirA recognition sequence (AviTag) *N*-terminally to the pVII open reading frame (ORF) such that the critical single nucleotide spacing to the upstream pV ORF and the start codon was maintained [15] (Fig. 1A). The M13K07 modified helper phage genomes were then introduced into host cells, propagated as normal helper phages and the culture supernatants titered for phage content as kanamycin resistant colony forming units (cfu^kanR^), which was found to be comparable to the non-pVII modified helper phage (Fig. S1A). Identical results were obtained with the pVII modified VCSM13 (*data not shown*). We then tested the modified M13K07 for their ability to rescue pIII display phagemids encoding either a single chain Fv (scFv) or a single chain T cell receptor (scTCR) [16]. The three pVII tagged helpers performed essentially as the untagged helper. Thus, the phage clearly tolerated peptides that differed in length, pI and charge fused *N*-terminally to pVII (Fig. S1B - D). We then absorbed virions to tag-specific supports (Fig. 1B-E). The AviTag-virions were absorbed to streptavidin (SA) coated beads, and bound virions detected by an anti-M13-HRP monoclonal antibody (mAb). Initial experiments demonstrated inefficient virion capture, presumably due to low endogenous BirA activity as the standard phagemid packaging protocol is conducted at 30°C (*data not shown*). This was overcome by making a new F^+^ *E. coli* host strain, AVB100FmkII, which over-expresses *birA*. Phagemid rescue using this strain led to markedly improved SA-specific binding of the virions (Fig. 1B, *left part*). Approximately 70% of the input phages were captured on beads estimated by titering the phage content in the waste (as cfu^ampR^) after extensive washing of the bead-virion complexes (Fig. 1B, *right part*). These data demonstrate that the AviTag-pVII fusion is surface exposed and functional as a BirA substrate. The FLAG-tagged phages bound strongly to two different anti-FLAG mAbs, M2 and M5 (Fig. 1C). M5 binds the FLAG peptide only when located at an unprocessed *N*-terminus, and the strongest reactivity was seen with M5, nearly reaching the level observed with polyclonal anti-pVIII antibodies. The latter was surprising, since phages contain thousands of pVIII epitopes and a maximum of only five pVII-FLAG epitopes. Thus, the observed binding clearly underscores strong and specific M5 phage absorption. The HIS_6_-tagged virions bound IMAC matrix beads in a pH dependent manner (Fig. S2), and the binding could be specifically abolished using imidazole (Fig. 1D). Increasing the spacer length between pVII and the HIS_6_-tag did not increase the efficiency of bead capture (Fig. S2). Moreover, similar IMAC bead capture properties was also seen with pVIII display phagemid virions rescued using this HIS_6_-pVII helper phage system (Fig. 1E, *left part*), and based on the in-put to out-put titers after elution, there was a 150-fold differential recovery depending on the absence or presence of the HIS_6_-modification (Fig. 1E, *right part*). Imaging analysis by electron microscopy showed that the pVII modified virions were morphologically identical to the unmodified virions (*data not shown*), ruling out that the pVII modification introduces any gross physical alterations of the virions as compared to their unmodified counterparts.

### pVII-tagging differentiate mixed virion populations

**Figure 2:**
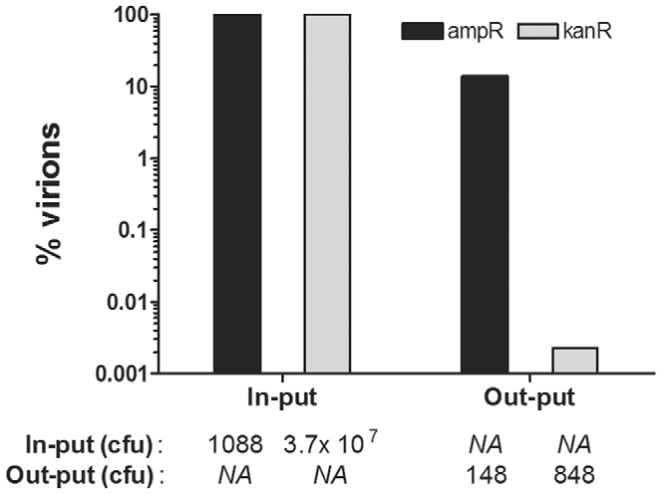
Tag-pVII mediated enrichment of virions. *In vivo* biotinylated phagemid (ampR) virions displaying a scTCR on pIII and AviTag on pVII were spiked into VCSM13 helper phage (kanR) at a ratio of approximately 3×10^−5^. After a single round of SA bead capture and extensive washing, the amount of phagemid (cfu^ampR^) and helper phage (cfu^kanR^) virions captured were determined using the beads for direct infection as described in *Methods*. The results are shown as percent of in-put retrieved (*NA*, not applicable).

The ability to produce distinct virion populations with different pVII tags may allow their physical separation from complex mixtures. As proof of concept, we blended a small amount of *E. coli* AVB100FmkII *in vivo* biotinylated AviTag-virions into a large amount of untagged “background“ virions. We then did a single SA bead capture, and the bead-virion complexes used for direct infection of fresh host cells after extensive washing. We found that the procedure led to a 5×10^3^ fold enrichment of tagged virions with an untagged background of 0.002% (Fig. 2).

### Bispecific multivalent pIII and pVII display

The results above clearly show that bispecific phages are obtained by the combined use of pIII (phagemid encoded) and pVII (helper phage encoded) display. To test whether virions are assembled when pIII and pVII fusions are encoded within the same phage genome, we introduced the AviTag-pVII modification and the scTCR-pIII fusion into fUSE5 [17], thereby creating the bispecific pIII-pVII phage genome display vector f37^AviTag^. Functional display on both distal tips of the virions was indeed demonstrated (Fig. S3).

## Discussion

Heterologous peptide display on pIII or pVIII is based on signal sequence dependent translocation of the fusion from cytosol to the periplasm. Here, we show efficient display on another capsid protein, namely pVII. In contrast to earlier reports, this was achieved without leader mediated periplasmic targeting of the fusion [14], [18]. Despite the fact that the pVII protein lacks any known signal sequence, it is yet inserted into and spans the inner membrane prior to virion incorporation without post-translational processing [4], [19]. Although the mechanism of both wt and modified pVII membrane insertion is unknown, the lack of a signal sequence signature suggests a different transport route than either of the SRP-, SEC, TatABC or YidC-dependent pathways of the *E. coli* secretory machinery [20]. The discrepancy between the current and an earlier report regarding the feasibility of genomic pVII fusion protein display without an *N*-terminal leader sequence may at least partly be due to the nature of the chosen fusion protein [19]. The former study used glutathione-S-transferase, which is a globular protein that readily folds and dimerizes in the cytosol, which may have hampered periplasmic targeting [21]. The lack of an *N*-terminal leader sequence may alleviate unpredictable heterogeneity in functional peptide display due to incomplete or lack of leader peptidase signal sequence cleavage [22].

All three pVII-tag fusions reported herein were encoded on helper phage genomes, which in turn were used to support production of virions with heterologous fusions to pIII or pVIII encoded on phagemids. Thus, the resulting virions displayed a tag on pVII as well as larger fusions on pIII or pVIII. In contrast to earlier reports on bispecific display that requires reformatting to new dedicated vectors, these novel helper phages significantly improves the versatility and ease of use with existing systems [8], [9], [13], [18]. Any pIII or pVIII display phagemid encoding single clones or a library can be used with one of the pVII modified helper phages described, and bispecific display with a pVII-tag of choice is obtained after standard phagemid rescue. Importantly, the tag on pVII does not interfere with the function of the other fusion. Furthermore, the tag-peptides reported herein, namely AviTag, FLAG and HIS_6_, which are among the most commonly used tags for detection, immobilization and purification, may be very easily exchanged with another tag of interest in a quick helper phage mutagenesis step. As a very high affinity SA tag, AviTag was chosen, rather than alternatives such as the Strep-tag, despite its need for enzymatic modification to gain functionality. This potentially labor intensive step was easily integrated into the normal phage propagation cycle merely by exploiting an F positive version of the *birA* over-expressing *E. coli* strain AVB100. *In vivo* biotinylation of the virion was thereby achieved without adding extra processing steps.

In the examples above, the pIII and pVII fusions are found on separate genetic elements (a phagemid and a helper phage genome); hence only low valence pIII is achieved. Using the genome display vector f37, we also show that pIII and pVII fusions are equally well tolerated when encoded in the same vector rendering multivalent bispecific display. This genomic system also offers the potential advantage of simplifying and speeding up the turn over time during propagation as no helper phage super infection step in conjunction with controlled bacterial growth and processing steps are needed.

Finally, we show that one virion population can be retrieved after mixing with a different virion population, using the pVII fusion tag. Importantly, the separation of the virion particles takes place in solution before any selective propagation step. Differential tagging of e.g. two separate pIII libraries would allow library against library selection [23] and physically distinguish between two (or more) populations of virions independently of their POIs displayed.

Although not explored here, it is highly conceivable that type 7 offers an attractive alternative to current type 3 display for construction of and affinity selection from large peptide libraries [24]. Since pIII is of particular importance for early events in *E. coli* entry, a complete lack of infectivity interference is expected when pIII is wt [25]. Indeed, in a direct comparison between signal sequence-dependent genomic pIII and pVII display, Kwasnikowski *et al*. reported superior antigen reactivity with pVII display [14]. It may well be that signal sequence independent peptide libraries offer an additional advantage due to lack of heterogeneous leader peptidase processing [22]. An additional advantage to pVII display, leaving pIII unaltered, is that virion rescue following a library selection step may be performed without breaking the virion-target bond, as elution may be done by infection directly on the solid phase. Especially for retrieving high affinity binders, when the strong virion-target interaction may be resistant to a variety of elution strategies, this simplifies rescue and may well increase the successful isolation of such binders [26].

**Figure 3:**
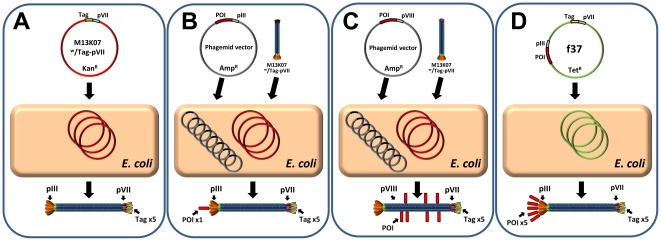
Tag-pVII phage display routes. (**A**) The M13K07 helper phage modified with an *N*-terminal pVII tag assembles into virions displaying the tag of choice on all 3–5 pVII copies on the virion tip. In principle, this modified helper phage genome is therefore analogous to a pIII phage genome vector. (**B**) Used in phagemid rescue of any standard pIII phagemid in a 3+3 system, the Tag-pVII modified helper phage yields defined, bispecific virions. Normally, such pIII phagemids render low valence display on pIII, whereas there will be multivalent Tag-pVII display. (**C**) Used in phagemid rescue of any standard pVIII phagemid in an 8+8 system, the Tag-pVII modified helper phage yields bispecific display where all pVII carry the tag, whereas the many thousand pVIII copies of the virion body will be a heterogeneous blend of POI-pVIII dispersed among wt pVIII. (**D**) When the Tag-pVII modification is integrated into a pIII phage genome display vector, a system is made in which both virion tips are fully modified with a fusion peptide on all copies of both pIII and pVII.

In summary, we have demonstrated display of three peptide tags on pVII that are up to 17 aa in length and have different charge and pI. Hence, despite the large heterogeneity in the physiochemical properties between these three fusions, no apparent influence on wt pVII function was observed. A summary of the different phagemid and phage genome display methods are shown in Figure 3. We believe this pVII-tagging approach by use of modified helper phages will significantly expand the versatility of existing pIII and pVIII display.

## Materials and Methods

### Plasmids, bacterial strains, phage and materials

The phOx-BSA and pSEX81 phagemid vector harboring a phOx-BSA specific affinity matured human scFv was kindly provided by Affitech Research AS (Oslo, Norway). The pSEX and pFKPDN phagemids harboring the scTCR Vαβ4B2A1, as well as the phage genome vectors fUSE5-scTCR Vαβ4B2A1 and pSC have been described previously [16], [27]. The *E. coli* strains K91K (*thi, lacZ^-^*:Tnλ*NK1105* (Kan^r^)) and MC1061 (*hsdR mcrB araD139 Δ(araABC-leu)7679Δ(lac)174 galU galK strA thi)* were obtained from Dr. G. P. Smith (Division of Biological Sciences, University of Missouri, USA). The *E. coli* strains XL1-Blue (*recA1 endA1 gyrA96 thi-1 hsdR17 supE44 relA1 lac* [F *proAB lacI* ^q^ *ZΔM15* Tn*10* (Tet^r^)] and CJ236 (*FΔ(HindIII)::cat (Tra+Pil+Cam^r^)/ung-1 relA1 dut-1 thi-1 spoT1 mcrA*) were purchased from Stratagene (LaJolla, CA, USA) and New England Biolabs (Ipswich, MA, USA), respectively. The *E. coli* MC1061-derived AVB100 strain that harbors an L-arabinose inducible *birA* cassette was purchased from Avidity LLC (Aurora, CO, USA). To obtain the F-positive AVB100FmkII (Str^r^/Tet^r^), the AVB100 parent was mated with XL1-Blue using standard methodology [2]. Likewise, the F-positive AVB100F (Str^r^/Cam^r^) was obtained by mating of AVB100 with CJ236. M13K07 and VCSM13 helper phages were purchased from GE Healthcare (Uppsala, Sweden) and New England Biolabs (Ipswich, MA, USA), respectively. All restriction enzymes (RE) were purchased from New England Biolabs (Ipswich, MA, USA). DNA oligos were purchased from MWG Biotech AG (Ebersberg, Germany). Pfu Ultra and Phusion DNA polymerases were purchased from Stratagene (LaJolla, CA, USA) and Sigma-Aldrich (Oslo, Norway), respectively. The anti-M13-HRP Ab and anti-FLAG M2 and M5 mAbs were purchased from GE Healtcare (Uppsala, Sweden) and Sigma-Aldrich (Oslo, Norway), respectively. The F23.2 mAb was a kind gift from Dr. Uwe D. Staerz (Department of Medicine, National Jewish Medical and Research Center, Denver, USA) and the GB113 mAb [28] was purified from cell supernatant on protein G-sepharose (GE Healtcare, Uppsala, Sweden). Dynabeads TALON™ and MyOne™ were purchased from Invitrogen (Oslo, Norway). All media and buffers were prepared essentially as described [29].

### Design and construction of the pVII modified phage genomes

Codon-optimized versions for prokaryotic expression of AviTag™ (*N*-MSGLNDIFEAQKIEWHE-*C*), FLAG-tag (*N*-MDYKDDDDK-*C*) and the HIS_6_-tag (*N*-MHHHHHH-*C*) peptide sequences were attached *5′*-terminally to the pVII ORF in M13K07 by QuikChange™ *in vitro* mutagenesis according to the manufacturers' protocol (Stratagen, LaJolla, CA, USA). Primer design was based on a DNA sequence alignment of M13K07, VCSM13 and fUSE5 (Fig. S4). The modifications were verified by DNA sequencing (*in-house* ABI lab DNA sequencing core facility, Dept. Molecular Biosciences, University of Oslo). To ensure a clean vector background, a *BsrG*I/*SnaB*I RE fragment containing the modified pVII was moved into unmodified genomes on compatible RE sites using standard techniques. The DNA constructs were introduced into the *E. coli* hosts XL1-Blue or MC1061 by electroporation. Primer sequences and GenBank accession numbers for the resulting constructs are listed in Table S1.

### Virion production

Phagemid rescue from *E. coli* XL1-Blue using M13K07 or VCSM13 helper phages was done essentially as described [30]. Recombinant phages were amplified from *E. coli* MC1061 transformed with fUSE5 essentially as described [31]. Phagemid rescue in AVB100mkII and phage amplification in AVB100F were done essentially as for XL1-Blue and MC1061, respectively, and increased *in vivo* biotinylation was achieved by supplementing the growth medium with 50 µM d-biotin and 0.4% w/v L-arabinose (final concentrations). Virion assembly was monitored by spot titration as described [25]. Where applicable, the virions were purified and concentrated by PEG/NaCl precipitation as described [32], and resuspended in PBS, pH 7.4. To estimate the *in vivo* biotinylation level in AVB100mkII, the virions were 2x PEG precipitated and captured on SA beads as described below. The biotinylation level was then determined as 100-*f*, where *f* is the fraction (in %) of virions in the waste of the in-put.

### Phage capture ELISA and bead capture

In ELISA, the various targets (Abs and phOx-BSA) were absorbed to MaxiSorp™ microtiter plate wells (Nunc, Roskilde, Denmark) in concentrations from 2.5 to 5 µg/ml in PBS, pH 7.4 overnight at 4°C. The wells were blocked with either PBSTM (PBS supplemented with 0.05% v/v Tween 20 and 4% w/v skim milk) or 2% w/v BSA (in PBS) for 1 h at room temperature (RT). Virion preparations where then added and allowed to react for 1 to 2 h at RT, before captured virions were detected with anti-M13-HRP (1∶5,000) for 1 h at RT. The wells were developed with ABTS substrate and the absorbance read at A_405 nm_. Alternatively, the wells were developed with TMB soluble (Merck KGaA, Darmstadt, Germany), stopped with 1M HCl, equilibrated and the absorbance read at A_450 nm_.

For AviTag phage bead capture, 50 µl/tube MyOne™ T1 beads were transferred to fresh 1.5-ml tubes and 500 µl 2% BSA in PBS (w/v) was added. Likewise, 250 µl of virion containing cleared supernatant were transferred to 1.5-ml tubes and supplemented with 250 µl of 2% BSA. The tubes were then incubated for 1 h at RT on a rotating wheel. Thereafter, the beads were washed 3x by first immobilizing the beads by using a Dynal tube magnet rack. The supernatant was discarded and 0.5 ml of PBST (PBS supplemented with 0.05% Tween 20) added to each tube. The tubes were taken out of the rack and briefly vortexed before re-entered into the rack. The supernatant was again cleared and the washing repeated twice. The tubes were removed from the rack and 250 µl of blocked phage and 250 µl PBST were added to each tube. The tubes were then incubated for 1.5 h/RT on a rotating wheel. The tubes were washed 3x in PBST as described above. 0.5 ml of PBST containing an anti-M13-HRP Ab (1∶2000) was then added to each tube and the tubes were incubated for 1 h/RT on a rotating wheel. The tubes were washed 3x in PBST as described above. 500 µl TMB soluble was added and the reaction stopped by an equal volume of 1M HCl after 5 min at RT. 100 µl of each solution was transferred to Maxisorp ELISA strips (Nunc, Roskilde, Denmark) and the absorbance measured at A_450 nm_. For IMAC phage bead capture, 50 µl/sample TALON™ beads were prepared in Washing and Binding (WB) buffer, either with pH 7 or 8, essentially as described by the manufacturer. In one experiment, the WB buffer (pH 8) was supplemented with 300 mM imidazole. The appropriate phage samples were then added in a total volume of 500 µl (supernatant or PEG precipitated) and incubated for 1 h/RT on a rotating wheel. After washing the beads as described above using WB buffer, anti-M13-HRP Ab (diluted 1∶5000 in PBST) was added to each tube and the tubes were further incubated 1 h/RT on a rotating wheel. After washing in PBST, 500 µl TMB soluble was added and the reaction stopped by an equal volume of 1M HCl after 5 min at RT. 100 µl of each solution was transferred to Maxisorp ELISA strips and the absorbance measured at A_450 nm_.

## Supporting Information

Figure S1 Virion assembly and functionality analysis of pVII modified helper phages. (A) Virion assembly efficiency in E. coli XL1-Blue. Normal (denoted as wt) and pVII tag-modified M13K07 helper phage production was assessed by infectious titration, and values given as the number of kanamycin-resistant (kanR) colony forming units (cfu) per ml culture supernatant. (B) Phagemid rescue ability of normal and modified M13K07 helper phages. Ampicillin-resistant (ampR) anti-phOx scFv- and scTCR-pIII encoding phagemids were rescued and the virion production capacity given as the number of cfuampR/ml by infectious titration. (C) Phagemid (ampR) to helper phage (kanR) ratio in infectious titration. (D) The integrity of the pIII displayed POI (scFv anti-phOx or scTCR) on virions rescued with normal or the modified M13K07 helper phages assessed by antigen-specific phage capture ELISA. Briefly, phOx-BSA or the TCR surrogate Ag mAb GB113 (the cognate Ag for the TCR is the murine pMHC II complex I-Ed/λ2315) was coated in microtiter wells and interacting virions detected with an anti-M13 mAb as described in Methods. Notably, the GB113 Ab is clonotypic for the 4B2A1 TCR from which the scTCR is derived [28].
(0.28 MB TIF)

Figure S2 IMAC bead capture efficiency of HIS6-pVII virions depend on tag-pVII spacer and pH during capture. (A) PEG precipitated VCSM13 helper phage virions or pFKPDN-scTCR 4B2A1 phagemid-derived virions were prepared as described in Methods. The bead capture was done as described by the manufacturer protocol in the recommended sample volumes as described in Methods. The Binding and Washing buffer was used with either pH 7 or 8 and the virion in-put titer was normalized to 1x 1010/ml in PBS for all samples. Binding and subsequent detection of captured virions were done with an anti-M13 Ab as described in Methods. Two different HIS6-pVII modified helper phages were used, denoted HIS6 and HIS6-GS2, of which the latter has a 10 aa spacer between the HIS6-tag and the pVII capsid protein (illustration in B). Both versions of the HIS6-modified VCSM13 helper phage virions, but not the unmodified (denoted wt), were captured on the IMAC beads. The HIS6 version bound with higher efficiency than the HIS6-GS2 version. Moreover, virion capture was most efficient when done at pH 8. The latter was also seen with phagemid-derived virions packaged with the HIS6-pVII modified VCSM13 helper phage.
(0.22 MB TIF)

Figure S3 Bispecific multivalent scTCR-pIII and AviTag-pVII display encoded by a single phage genome. (A) Virion assembly efficiency in E. coli MC1061 from fUSE5 (harboring a scTCR 4B2A1 pIII fusion) and its pVII tag-modified derivative f37AviTag. Numbers of tetracycline-resistant (tetR) cfu/ml in culture supernatants and the corresponding PEG precipitated samples are given as assessed by infectious titration in E. coli K91K. (B) The integrity of the scTCR-pIII fusion on the virions assessed by binding to conformation (F23.2) and TCR clonotypic (GB113) mAbs in ELISA using titer normalized virion in-puts. (C) AviTag-pVII functionality as endogenous BirA substrate (both E. coli XL1-Blue (M13K07) and MC1061 (f37AviTag) assessed by virion binding to magnetic SA beads detected by an anti-M13 Ab. (D) Increased AviTag-pVII in vivo biotinylation is obtained with f37AviTag using the E. coli AVB100F host strain (see Methods) which over-expresses birA upon L-arabinose induction, as shown by increased virion binding to magnetic SA beads detected by an anti-M13 Ab.
(0.52 MB TIF)

Figure S4 Multiple sequence alignment of the M13K07, VCSM13 and fUSE5 genomes. The M13K07 (New England Biolabs sequence), VCSM13 (GenBank accession no.: AY598820) and fUSE5 (GenBank accession no.: AF218364) were aligned using ClustalX 2.0.5 and manually annotated using GeneDoc (http://www.psc.edu/biomed/genedoc). Only the relevant parts of the genomes framed by the unique BsrGI and SnaBI RE sites are shown together with the pV, pVII and pIX ORFs (only partial ORFs for pV and pIX, respectively).
(0.92 MB TIF)

Table S1 GenBank accession numbers and QuikChange mutagenesis primers. The M13K07 (New England Biolabs sequence: http://www.neb.com) and VCSM13 (GenBank accession no.: AY598820) is functionally identical both in their normal and pVII-modified versions. Both genomes are 100% identical in the BsrGI/SnaBI defined region where the pVII modification is found. The pVII modifications use were made in the M13K07 genome and verified sequences shuffled into VCSM13 and fUSE5 (thereby creating f37) on the compatible BsrGI/SnaBI RE sites. The sequences submitted to GenBank were based on the existing VCSM13 and fUSE5 GenBank entries AY598820 and AF218364, respectively.
(0.06 MB DOC)
