# Supplementary figures and images for: Expanding the Versatility of Phage Display I: Efficient Display of Peptide-Tags on Protein VII of the Filamentous Phage
Source: PLoS One. 2011 Feb 24;6(2):e14702. doi: 10.1371/journal.pone.0014702 (PMC3044727; doi:10.1371/journal.pone.0014702)

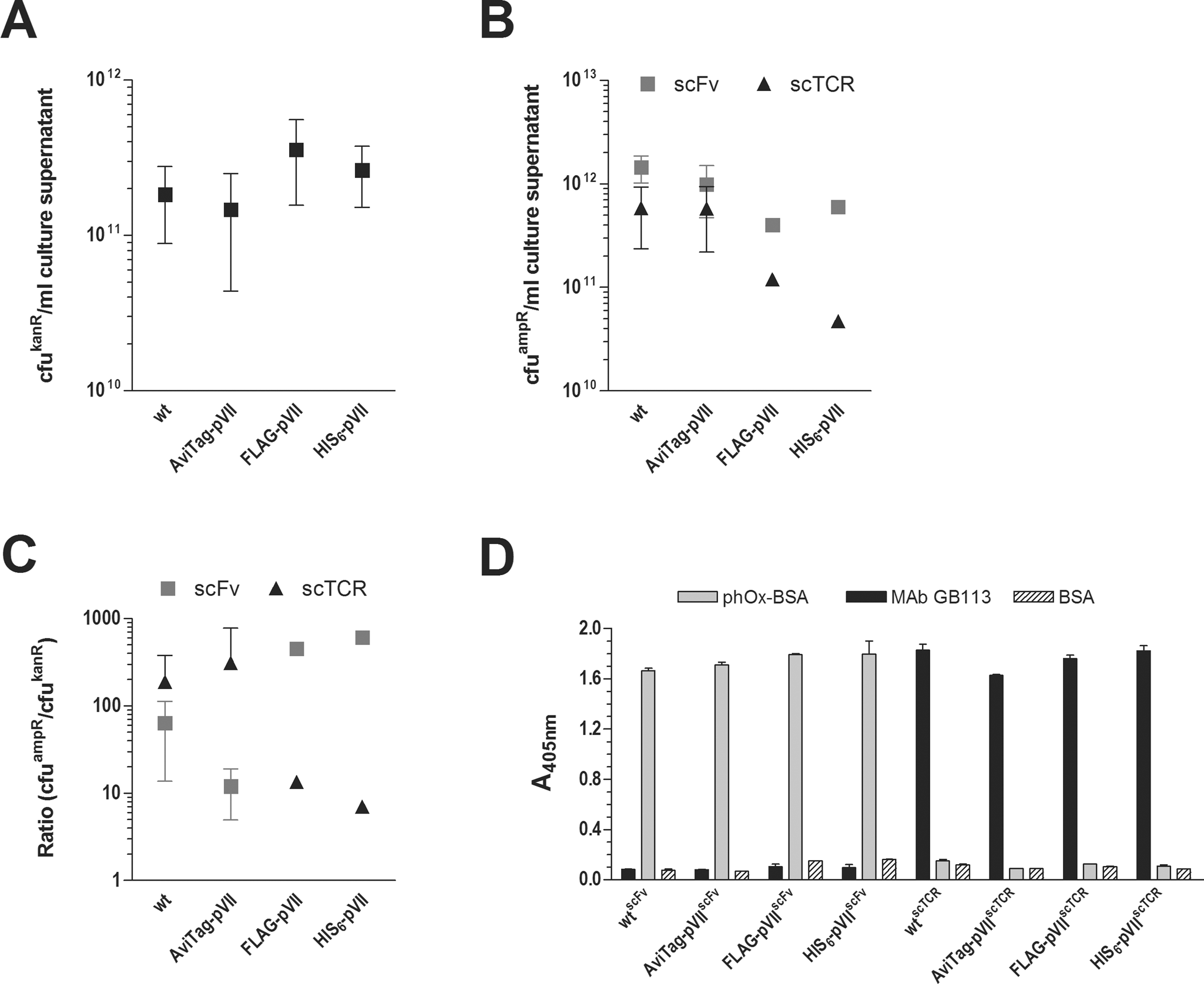

Supplement: Figure S1 — Virion assembly and functionality analysis of pVII modified helper phages. (A) Virion assembly efficiency in E. coli XL1-Blue. Normal (denoted as wt) and pVII tag-modified M13K07 helper phage production was assessed by infectious titration, and values given as the number of kanamycin-resistant (kanR) colony forming units (cfu) per ml culture supernatant. (B) Phagemid rescue ability of normal and modified M13K07 helper phages. Ampicillin-resistant (ampR) anti-phOx scFv- and scTCR-pIII encoding phagemids were rescued and the virion production capacity given as the number of cfuampR/ml by infectious titration. (C) Phagemid (ampR) to helper phage (kanR) ratio in infectious titration. (D) The integrity of the pIII displayed POI (scFv anti-phOx or scTCR) on virions rescued with normal or the modified M13K07 helper phages assessed by antigen-specific phage capture ELISA. Briefly, phOx-BSA or the TCR surrogate Ag mAb GB113 (the cognate Ag for the TCR is the murine pMHC II complex I-Ed/λ2315) was coated in microtiter wells and interacting virions detected with an anti-M13 mAb as described in Methods. Notably, the GB113 Ab is clonotypic for the 4B2A1 TCR from which the scTCR is derived [28]. (0.28 MB TIF) [file pone.0014702.s001.tif]

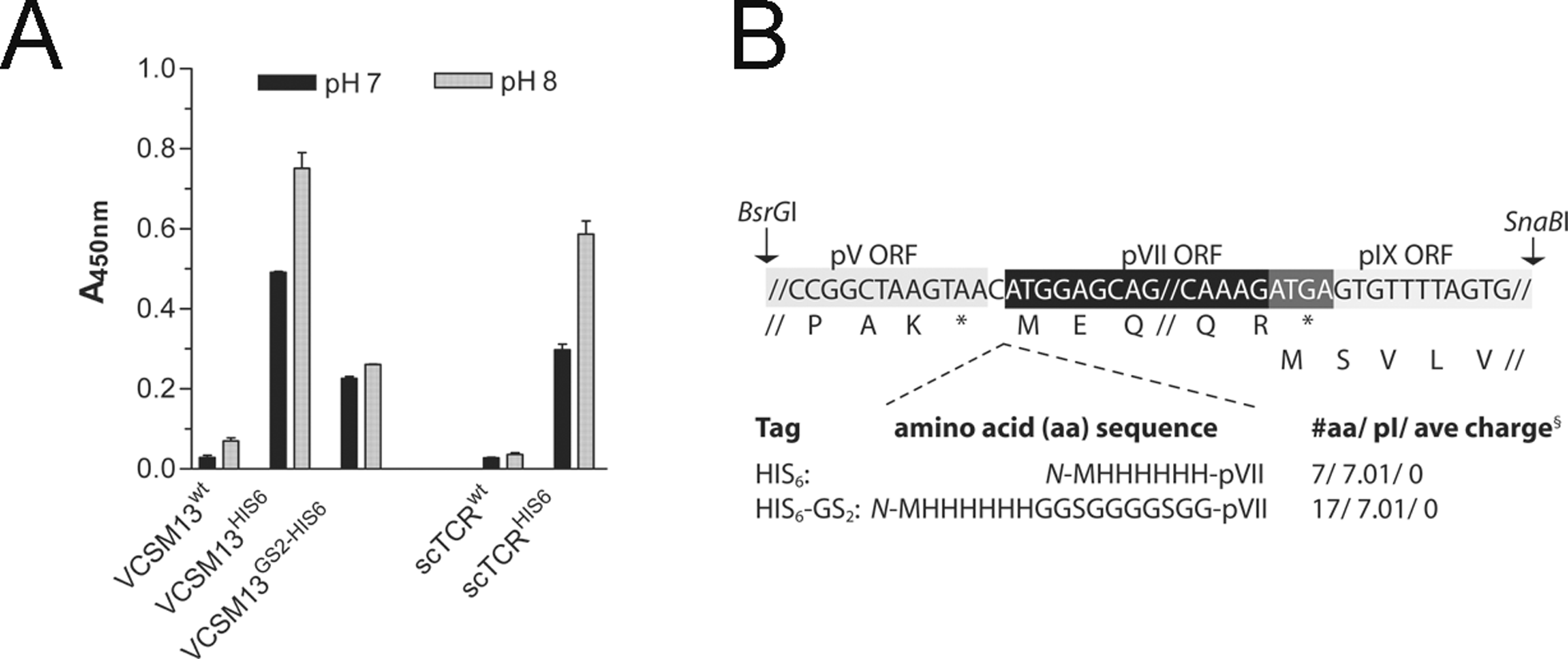

Supplement: Figure S2 — IMAC bead capture efficiency of HIS6-pVII virions depend on tag-pVII spacer and pH during capture. (A) PEG precipitated VCSM13 helper phage virions or pFKPDN-scTCR 4B2A1 phagemid-derived virions were prepared as described in Methods. The bead capture was done as described by the manufacturer protocol in the recommended sample volumes as described in Methods. The Binding and Washing buffer was used with either pH 7 or 8 and the virion in-put titer was normalized to 1x 10^10^/ml in PBS for all samples. Binding and subsequent detection of captured virions were done with an anti-M13 Ab as described in Methods. Two different HIS6-pVII modified helper phages were used, denoted HIS6 and HIS6-GS2, of which the latter has a 10 aa spacer between the HIS6-tag and the pVII capsid protein (illustration in B). Both versions of the HIS6-modified VCSM13 helper phage virions, but not the unmodified (denoted wt), were captured on the IMAC beads. The HIS6 version bound with higher efficiency than the HIS6-GS2 version. Moreover, virion capture was most efficient when done at pH 8. The latter was also seen with phagemid-derived virions packaged with the HIS6-pVII modified VCSM13 helper phage. (0.22 MB TIF) [file pone.0014702.s002.tif]

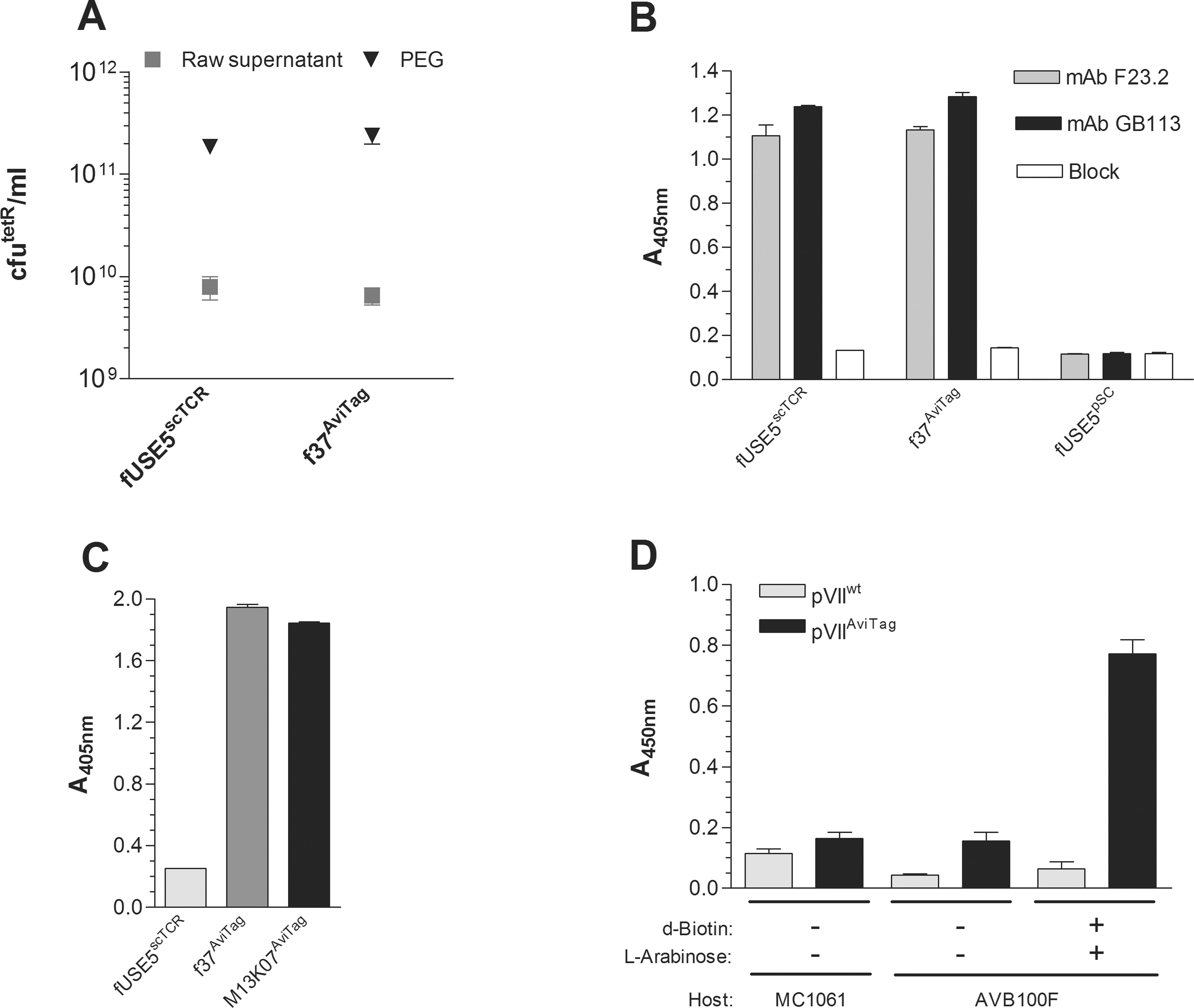

Supplement: Figure S3 — Bispecific multivalent scTCR-pIII and AviTag-pVII display encoded by a single phage genome. (A) Virion assembly efficiency in E. coli MC1061 from fUSE5 (harboring a scTCR 4B2A1 pIII fusion) and its pVII tag-modified derivative f37AviTag. Numbers of tetracycline-resistant (tetR) cfu/ml in culture supernatants and the corresponding PEG precipitated samples are given as assessed by infectious titration in E. coli K91K. (B) The integrity of the scTCR-pIII fusion on the virions assessed by binding to conformation (F23.2) and TCR clonotypic (GB113) mAbs in ELISA using titer normalized virion in-puts. (C) AviTag-pVII functionality as endogenous BirA substrate (both E. coli XL1-Blue (M13K07) and MC1061 (f37AviTag) assessed by virion binding to magnetic SA beads detected by an anti-M13 Ab. (D) Increased AviTag-pVII in vivo biotinylation is obtained with f37AviTag using the E. coli AVB100F host strain (see Methods) which over-expresses birA upon L-arabinose induction, as shown by increased virion binding to magnetic SA beads detected by an anti-M13 Ab. (0.52 MB TIF) [file pone.0014702.s003.tif]

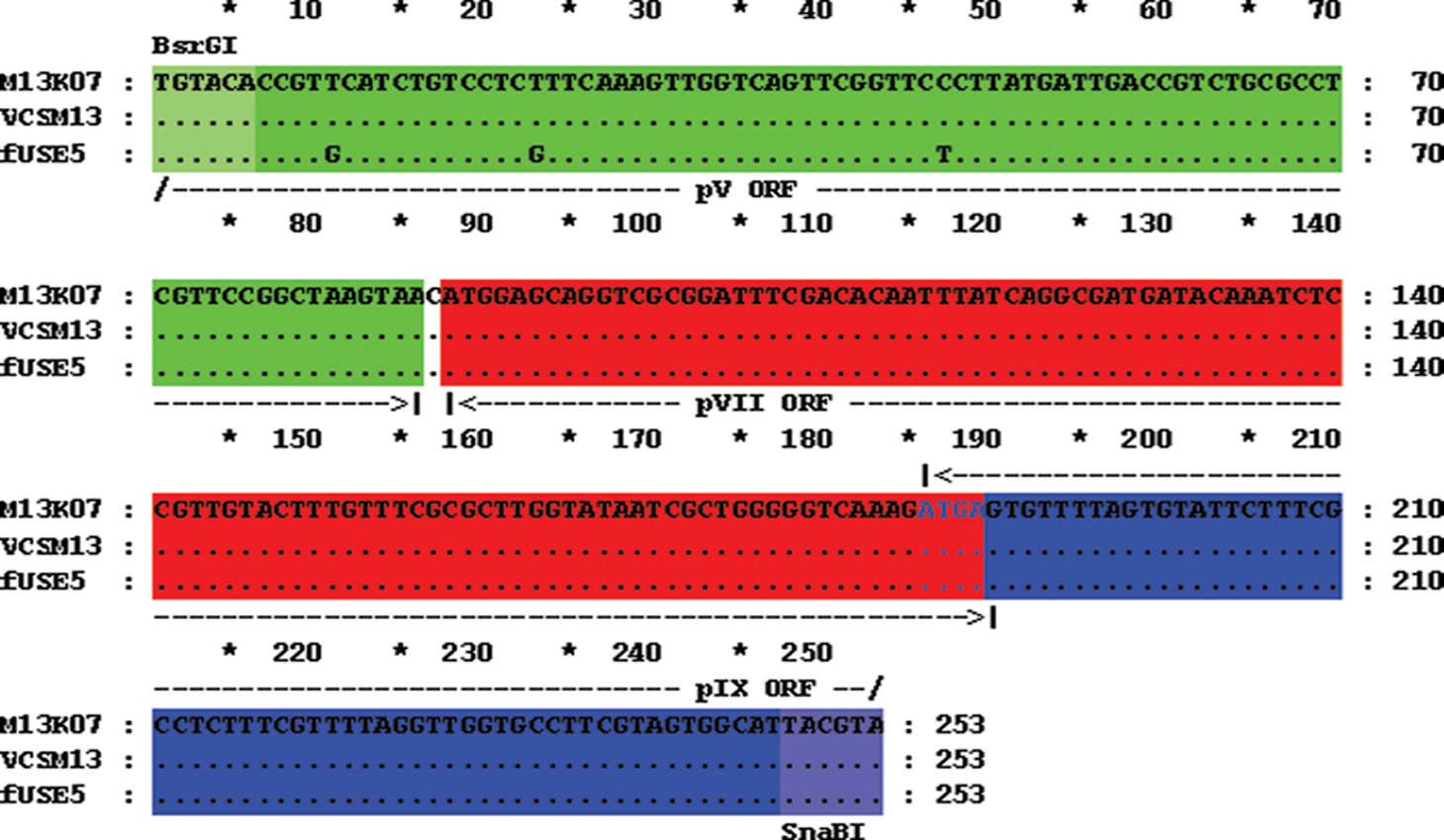

Supplement: Figure S4 — Multiple sequence alignment of the M13K07, VCSM13 and fUSE5 genomes. The M13K07 (New England Biolabs sequence), VCSM13 (GenBank accession no.: AY598820) and fUSE5 (GenBank accession no.: AF218364) were aligned using ClustalX 2.0.5 and manually annotated using GeneDoc (http://www.psc.edu/biomed/genedoc). Only the relevant parts of the genomes framed by the unique BsrGI and SnaBI RE sites are shown together with the pV, pVII and pIX ORFs (only partial ORFs for pV and pIX, respectively). (0.92 MB TIF) [file pone.0014702.s004.tif]
