# Supplementary material for: Expanding the Versatility of Phage Display I: Efficient Display of Peptide-Tags on Protein VII of the Filamentous Phage
Source: PLoS One. 2011 Feb 24;6(2):e14702. doi: 10.1371/journal.pone.0014702 (PMC3044727; doi:10.1371/journal.pone.0014702)
Supplement: Table S1 — GenBank accession numbers and QuikChange mutagenesis primers. The M13K07 (New England Biolabs sequence: http://www.neb.com) and VCSM13 (GenBank accession no.: AY598820) is functionally identical both in their normal and pVII-modified versions. Both genomes are 100% identical in the BsrGI/SnaBI defined region where the pVII modification is found. The pVII modifications use were made in the M13K07 genome and verified sequences shuffled into VCSM13 and fUSE5 (thereby creating f37) on the compatible BsrGI/SnaBI RE sites. The sequences submitted to GenBank were based on the existing VCSM13 and fUSE5 GenBank entries AY598820 and AF218364, respectively. (0.06 MB DOC) [file pone.0014702.s005.doc]

**Table S1.** GenBank accession numbers and QuikChange™ mutagenesis primers.

| **Construct** | **GenBank accession no.** | **Primer ID** | **Primer sequence (5’-3’)** |
| --- | --- | --- | --- |
| M13K07 w/AviTag-pVII | HQ592778 | BirA-pVII_frwd | CCGGCTAAGTAACATGTCCGGCCTGAACGATATCTTTGAAGCGCAGAAAATTGAATGGCATGAAATGGAGCAGGTC |
|  |  | BirA-pVII_rev | GACCTGCTCCATTTCATGCCATTCAATTTTCTGCGCTTCAAAGATATCGTTCAGGCCGGACATGTTACTTAGCCGG |
| M13K07 w/FLAG-pVII | HQ592779 | FLAG-pVII-frwd | CCGGCTAAGTAACATGGACTACAAAGATGACGATGACAAAATGGAGCAGGTCG |
|  |  | FLAG-pVII-rev | CGACCTGCTCCATTTTGTCATCGTCATCTTTGTAGTCCATGTTACTTAGCCGG |
| M13K07 w/HIS_6_-pVII | HQ592780 | HIS6-pVII-frwd | CCGGCTAAGTAACATGCATCACCATCACCATCACATGGAGCAGGTCG |
|  |  | HIS6-pVII-rev | CGACCTGCTCCATGTGATGGTGATGGTGATGCATGTTACTTAGCCGG |
| f37^AviTag^ | HQ592781 |  |  |
